# Innovative Approaches for Crop Improvement and Sustainable Management of Plant Disease in the Post-Genomic Era

**DOI:** 10.3390/ijms23063273

**Published:** 2022-03-18

**Authors:** Marco Fambrini, Claudio Pugliesi, Susanna Pecchia

**Affiliations:** 1 Department of Agriculture Food and Environment, University of Pisa, Via del Borghetto 80, 56124 Pisa, Italy; marco.fambrini@unipi.it (M.F.); susanna.pecchia@unipi.it (S.P.); 2 Interdepartmental Research Center Nutrafood “Nutraceuticals and Food for Health”, University of Pisa, Via del Borghetto 80, 56124 Pisa, Italy

Safeguarding food supply in a world environment subject to sudden climate change, reducing the use of anthropogenic sources of pollution as much as possible, and using crops that must necessarily be increasingly resilient to biotic and abiotic stresses is a mandatory and ambitious necessity for the foreseeable future. To achieve this goal, modern biotechnological solutions are called for in order to become our decisive tools. In this context, our Special Issue aimed to call original research contributions and/or critical reviews from the available literature, which focus their attention on the genetic improvement and the protection of plants against pathogens with new solutions enabled by the genome sequencing revolution.

In this Editorial, we revisit the eight peer-reviewed papers included in the Special Issue: five articles and three reviews. As for the articles, three focused on understanding the interaction between crops and pathogenic fungi. In the first [1], accessions of *Triticum turgidum* were tested for resistance to *Blumeria graminis* and genotyped with a single nucleotide polymorphism array to identify new sources of resistance genes. In the second [2], a chestnut resistance gene was transferred to *Quercus suber* with an *Agrobacterium*-mediated technique to induce tolerance to *Phytophthora cinnamomi*. The third article [3] deals with exogenous dsRNA treatments in lettuce to determine the silencing of the *Botrytis cinerea Bmp3* gene because it plays an important role in the expression of pathogenicity. In the article by Batool et al. [4], two entomopathogenic fungi were used in the *Zea mays*–*Ostrinia furnacalis* interaction to perform an integrated pest management strategy. In the fifth article, Edgü et al. [5] aimed to develop a rapid, efficient, and repeatable technique for detecting tobacco rattle virus infections in *Solanum tuberosus* for use outside a laboratory. The Special Issue has also been enriched by three reviews: (i) identification of candidate resistance gene analogues in the *Brassica* genus, exploiting genome assembly [6], (ii) recent biotechnological solutions in order to counteract harmful fungi and oomycetes in grapevines [7], and (iii) increasing tolerance towards the main abiotic stresses in crops through unconventional technologies applicable to genetic improvement [8].

Therefore, each paper will be presented in more detail below.

The work of plant breeders can be assisted by the identification of genes for the resistance to common pathogens. Moreover, plants with resistance traits are useful for limiting the use of pesticides. In this context, Simeone et al. [1] presented the identification of novel quantitative trait loci (QTLs) for resistance to *Blumeria graminis* (usually known as powdery mildew) in wheat. The authors identified five QTLs for adult plant resistance and three QTLs for seedling resistance mapped on chromosome regions where no resistance gene had previously been reported. The plant material analysed was a collection of wild and cultivated tetraploid wheats (*Triticum turgidum*), of which *Blumeria graminis* (DC) Speer f. sp. *tritici* Em. Marchal (syn. *Erysiphe graminis* f. sp. *tritici*) (Btg) is a pathogenic fungus of primary importance. In particular, Simeone et al. [1] underlined the genetic diversity for seedling resistance (SR) and adult plant resistance (APR) to powdery mildew in wild and cultivated tetraploid wheats comprising seven subspecies of *Triticum turgidum* (*durum*, *turanicum*, *polonicum*, *turgidum*, *carthlicum*, *dicoccum* and *dicoccoides*) by exploiting genomic resources and single nucleotide polymorphism (SNP) markers. The major aims were (i) to perform a genome-wide association study (GWAS) to identify novel sources of powdery mildew resistance genes, (ii) to identify the exact map position of associate SNP markers on the high-density SNP-based consensus *T. durum* map, and (iii) to ascertain candidate genes. Notably, the marker IWB6155, associated with the APR QTL *QPm.mgb-1AS* at 10.8 cM on chromosome arm 1AS, was positioned within the *TRITD1Av1G004560* gene, encoding a disease resistance protein RPM1. Two other SNP markers, IWB42940 and IWB35735, linked to the QTL *QPm.mgb-6BL.3* and *QPm.mgb-7AL* for SR, were identified as closed (3 and 10 Kb) to the *TRITD6Bv1G207200* and *TRITD7Av1G27148*0 genes encoding FBD-associated F-box protein and Pm3-like disease resistance protein, respectively. The results obtained from tetraploid wheat accessions are very useful because this material can be easily crossed with cultivated common and durum wheat in order to increase and diversify the source of *Bgt* resistance genes. Indeed, as proposed by the authors [1], SNP markers closely linked to powdery mildew resistance QTL/genes can be used directly or transformed into highly specific and sensitive kompetitive allele specific PCR (KASP) markers by reducing the time required in marker-assisted selection programs.

Traditional plant breeding programs in tree crops are often hampered by the long-life cycle of these plants. A biotechnological solution able to significantly reduce the time needed for this outcome could be the *Agrobacterium*-mediated approach to modify tree genomes. Unfortunately, these programs are often inapplicable because the in vitro regenerative potential is very low in trees when compared to that of model plants such as *Arabidopsis thaliana*. Therefore, the research by Cano et al. [2] in *Quercus suber* (cork oak) is particularly appreciable since, for these authors, the fundamental goal achieved was an effective and reproducible procedure to transform three genotypes of cork oak, selecting transformants with an improved response to *Phytophthora cinnamomi*, a pathogen highly destructive to the cork oak crops in European environments. To obtain these valuable genotypes, the trait selected was the expression of the chestnut *CsTL1* gene encoding a thaumatin-like protein, a pathogenesis-related protein produced by the host with strong antifungal activity. Cork oaks are an important tree for the production of cork with a very high economic value throughout the Iberian Peninsula and beyond. Unfortunately, the “la seca” syndrome has caused extensive damage in the most intensively cultivated agricultural areas and a probable key agent of this syndrome is *Phytophthora cinnamomi*. The authors isolated genotypic lines of *Q. suber* with high embryogenic potential from leaf explants and finalized the in vitro selection steps of the plant material actually transformed by *Agrobacterium* [2]. In addition, the authors also developed a cryopreservation treatment of transgenic cork oak somatic embryos useful for the preservation of the precious material. Compared to the cork oak transformation programs previously carried out, Cano et al. [2] were able to concretely assess the transformed plants obtained, evaluating the expression of the desired character. In particular, the authors demonstrated that transformed plants showed *P. cinnamomi* symptoms after inoculation with a significant delay compared to the wild type [2].

The exogenous application of dsRNA-mediated silencing is an RNA interference (RNAi) strategy and has been reported as a non-genetically modified organism (non-GMO) promising opportunities in plant defence against fungi and insects by targeting specific genes without the emission of pollutants. Spada et al. [3] chose to focus on silencing the *Botrytis cinerea Bmp3* (*BcBmp3*) gene encoding for a Slt2-MAP kinase. This protein is a key player involved in the pathogenic signalling of plants infected by *B. cinerea*. The uptake of RNAs from the environment, a phenomenon named environmental RNAi, was demonstrated in *B. cinerea*, which can take up both siRNAs and dsRNAs directly, inducing the silencing of the pathogen genes. Spada et al. [3] demonstrated that the silencing of the *BcBmp3* gene by the application of exogenous dsRNA affects fungal growth and virulence on *Lactuca sativa* leaves. *B. cinerea* attacks a remarkable number of crops with a significant loss of product. To counteract this destructive pathogen, nowadays it will be necessary to drastically reduce the traditional control solutions that exacerbate risks to human health and the environment. In this article, the effect of topical applications of dsRNA constructs was evaluated in vitro by a fungal growth assay in microplates and in vivo on artificially inoculated detached lettuce leaves. In both cases, topical applications of dsRNA led to *BcBmp3* knockdown with a delay in conidial germination, an evident growth retardation, and a strong reduction of necrotic lesions on the leaves. Interestingly, the authors explored the co-silencing effects of the *BcBmp3*-dsRNA construct. In silico off-target prediction and in vitro effects against the off-target fungus *Trichoderma harzianum* were performed and examined. In both cases, the *BcBmp3*-dsRNA construct was highly specific to *B. cinerea* with no off-target hits in the host plant, in distantly related phytopathogenic fungi, or in beneficial fungi or human genomes [3].

The containment of environmental pollution is a priority and a challenge to be overcome. Therefore, crop protection strategies that reduce pesticide use must be undertaken and possibly improved. *Ostrinia furnacalis* (the Asian corn borer, ACB) is an extremely harmful insect in *Zea mays* fields, especially in Asian environments. An interesting biological control strategy for dangerous pests can be the utilization of entomopathogenic fungi such as *Beauveria bassiana*. In this context, Batool et al. [4] analysed the synergistic effect of *Beauveria bassiana* and *Trichoderma asperellum* to elicit maize (*Zea mays* L.) defences against the Asian corn borer. This goal is part of the interesting perspective of mixing multiple biocontrol agents in order to enhance their potential. Amusingly, plants treated with these fungi greatly increase the activity of superoxide dismutase, which play a key role in the scavenging process of reactive oxygen species (ROS). Furthermore, a significant increase in proline, polyphenol oxidase, and protease activity was observed in infected plants [4]. The authors analysed the molecular aspects of the immune response based on the characterization of the *O. furnacalis* transcriptome after a fungal infection, using Illumina sequencing [4]. The results showed that the insect immune system becomes active against a *T. asperellum* attack, whereas *B. bassiana* alone and in combination with *T. asperellum* decreases the immune response, thus reducing ACB survival. Regarding the method of inoculation, seed coating or seed inoculation were the most effective in colonising plants, controlling *O. furnacalis*, and enhancing defence enzyme activities in maize plants compared to soil drenching. In conclusion, Batool et al. [4] demonstrated that the coculture of *B. bassiana* and *T. asperellum* has the synergistic potential to suppress the immune response of *O. furnacalis,* and this biological protection strategy can be very efficient in inducing plant resistance through the activation of defence-related enzymes.

In crops, viruses cause significant losses in production and quality, thus decreasing the marketable yield. Additionally, virus-associated losses in the fields are highly underestimated, as some viral infections are asymptomatic but synergistically contribute to damage from attacks by other pathogens. The reliability of molecular tests able to identify the presence of viral nucleic acids in crops is therefore a key point, especially if they are rapid, directly applicable in vivo, and accurate in ascertaining the presence of the virus. Edgü et al. [5] produced actual results on the identification of the tobacco rattle virus (TRV) in potatos. In particular, these authors developed a simple diagnostic method for the reliable detection of TRV without RNA purification, which involved minimal sample handling (mini), subsequent colorimetric loop-mediated isothermal amplification (LAMP), and final verification by lateral-flow dipstick (LFD) analysis. Notably, the tested mini-LAMP-LFD method greatly simplified sample processing and it did not require sophisticated laboratory tools. Moreover, the method generates results comparable in terms of precision and reliability to RT-PCR (sensitivity 89% and specificity 100%). Also noteworthy is the drastic reduction in the estimated cost when compared to the standard RT-PCR assay.

*Brassica* crops are economically very important because they are widely cultivated as oilseeds, vegetables, condiments, and forages. Unfortunately, several diseases hinder production in different environments and, therefore, the identification of candidate resistant gene analogues (RGAs) associated with disease resistance is essential for understanding disease mechanisms and management in breeding programs. Zhang et al. [6], carefully reviewed RGA identification in the *Brassica* genome and pangenome assemblies. After a preliminary introduction on the economical role of *Brassica* crops in the world, the authors retrieved the quantitative and qualitative disease resistance mechanisms in plants. In *Brassica*, the genome/pangenome-wide RGA prediction was also discussed. The identification of *Brassica* reference genome assemblies plays an important role in promoting QTL mapping and the discovery of candidate disease resistance genes. Predictable approaches to identifying causative RGAs are limited in their ability to correctly locate the causative gene. Indeed, QTL mapping often only allows for the identification of a large genomic region, which may contain multiple RGAs. However, genome-wide characterisation of RGAs using conserved domains and motifs in reference to genomes and pangenomes reveals their clustered arrangements and the presence of structural variations. In line with this, these authors reviewed the innovative RGASeq approaches to achieve efficient candidate identification. However, reference-based QTL mapping and cloning could be limited when exploring novel resistance in landraces and wild relatives which harbour greater genetic diversity. It is important to underline that the definition of the pan-RGAome will be essential to the understanding of the complex evolution of RGAs, which will provide insights on the mechanisms of disease resistance.

Grapevine is one of the most popular fruit crops with a prominent importance in agriculture. Unfortunately, the climatic requirements of *Vitis vinifera* are also particularly suitable for the deleterious development of pathogens such as downy mildew, powdery mildew, and grey mould caused respectively by *Plasmopara viticola*, *Erysiphe necator,* and *Botrytis cinerea*. However, in this fruit crop several biotechnological applications aimed at facilitating the achievement of genetic improvement objectives were indeed feasible. The review by Capriotti et al. [7] very effectively summarized the most recent biotechnological strategies optimized and applied to *Vitis* species, aimed at reducing their susceptibility to the most harmful fungal and oomycetes diseases. With respect to genetic engineering for the expression of candidate genes involved in fungal/oomycetes resistance, these authors presented an interesting table showing many representative examples of genetic transformation applied in *Vitis* species to improve resistance against the pathogens. The different attempts summarized in this table (Table 1 of [7]) show that various types of genes have so far been considered: the overexpression of transgenes encoding pathogenesis-related proteins, antimicrobial peptides, transcription factors, secondary stress-related metabolites, and defence-related proteins. Furthermore, a different strategy was provided to determine the gene silencing in *Vitis* through transformation with RNAi constructs or exogenous dsRNA treatments. However, to date, far fewer such experiments with two approaches have been performed on this fruit crop. In particular, the second strategy appears attractive because it could be a new type of treatment to reduce the environmental spread of pesticide pollutants. In grapevines, some useful reliable advances have recently been obtained with respect to some pathogens [9,10,11]. In grapevines, genome editing programs have been conducted for notable applications, and Capriotti et al. [7] reviewed these examples. Among these, authors also report cases in which more innovative attempts have been made as direct delivery of CRISPR/Cas9 ribonucleoproteins into protoplasts [12]. Interestingly, a knockout strategy was performed in grapevines with the CRISPR/Cas9 gene system encoding host susceptibility-related factors, to reduce the level of host damage caused by powdery mildew [13,14]. Finally, Capriotti et al. [7] included biosafety considerations and an overview of breeding technologies applied to enhance resistance against fungal and oomycetes diseases in grapevines. These aspects are very interesting because the development and application of traditional or innovative plant breeding strategies entail numerous technical advantages and disadvantages. Furthermore, considering that grapevines are used for human consumption, biosafety issues and public concerns must be carefully considered.

Preserving the productivity of the world's major crops even in the face of rapid climate changes and the growing demand for food is a daunting future challenge. Plants, sessile organisms by definition, must exploit their resilience to cope with the most common abiotic stressors such as extreme temperatures, water deficiency, anoxia due to submersion, excess of salts, or heavy metals. Obviously, under adverse climatic and/or edaphic conditions, survival may already be at risk and, therefore, the goal of preserving an acceptable production is even more problematic. In this Special Issue, Anwar and Kim [8] have contributed with a review that examines different aspects of plant response to stresses. The authors list modern biotechnological techniques to induce (or enhance) resistance to abiotic stress situations, providing many examples of research involving model plants and crops. The authors highlighted that the study of the physiology and biochemistry associated with abiotic stresses has returned a very complex picture in which the roles of individual genes are sometimes complex. However, this complexity makes it essential to characterize gene function as deeply as possible and, in this modern biotechnology, it can facilitate the main goal. Anwar and Kim overviewed the importance of QTL mapping for plant breeding programs [8]. Moreover, the authors discussed a current overview of identification, functions, and roles of miRNA in crops for abiotic stress tolerance, as well as examples for improving abiotic stress tolerance through overexpressing selected miRNAs. Then, Anwar and Kim [8] analysed how genome editing using CRISPR/Cas9 was used to improve plant resilience in adverse environmental conditions. In recent years, this has been a very active research field with interesting future prospects, especially in order to shorten the time for selecting the best plant material as much as possible.
